# Living in uncertainty due to floods and pollution: the health status and quality of life of people living on an unhealthy riverbank

**DOI:** 10.1186/s12889-018-5706-0

**Published:** 2018-06-22

**Authors:** Fredrick Dermawan Purba, Joke A. M. Hunfeld, Titi Sahidah Fitriana, Aulia Iskandarsyah, Sawitri S. Sadarjoen, Jan J. V. Busschbach, Jan Passchier

**Affiliations:** 1 Department of Psychiatry, Section Medical Psychology and Psychotherapy, Erasmus MC University Medical Center, Wytemaweg 80 Room Na2018, 3015 CN Rotterdam, The Netherlands; 2 Department of Developmental Psychology, Faculty of Psychology, Padjadjaran University, Jatinangor, Indonesia; 3 Faculty of Psychology, YARSI University, Jakarta, Indonesia; 4 Department of Clinical Psychology, Faculty of Psychology, Padjadjaran University, Jatinangor, Indonesia; 5 Department of Clinical, Neuro & Developmental Psychology, VU University, Amsterdam, The Netherlands

**Keywords:** Quality of life, Health status, Happiness, Life satisfaction, Water pollution, Indonesia

## Abstract

**Background:**

People living on the banks of polluted rivers with yearly flooding lived in impoverished and physically unhealthy circumstances. However, they were reluctant to move or be relocated to other locations where better living conditions were available. This study aimed to investigate the health status, quality of life (QoL), happiness, and life satisfaction of the people who were living on the banks of one of the main rivers in Jakarta, Indonesia, the Ciliwung.

**Methods:**

Respondents were 17 years and older and recruited from the Bukit Duri community (*n* = 204). Three comparison samples comprised: i) a socio-demographically matched control group, not living on the river bank (*n* = 204); ii) inhabitants of Jakarta (*n* = 305), and iii) the Indonesian general population (*n* = 1041). Health status and QoL were measured utilizing EQ-5D-5L, WHOQOL-BREF, the Happiness Scale, and the Life Satisfaction Index. A visual analogue scale question concerning respondents’ financial situations was added. MANOVA and multivariate regression analysis were used to analyze the differences between the Ciliwung respondents and the three comparison groups.

**Results:**

The Ciliwung respondents reported lower physical QoL on WHOQOL-BREF and less personal happiness than the matched controls but rated their health (EQ-5D-5L) and life satisfaction better than the matched controls. Similar results were obtained by comparison with the Jakarta inhabitants and the general population. Bukit Duri inhabitants also perceived themselves as being in a better financial situation than the three comparison groups even though their incomes were lower.

**Conclusions:**

The recent relocation to a better environment with better housing might improve the former Ciliwung inhabitants’ quality of life and happiness, but not necessarily their perceived health, satisfaction with life, and financial situations.

## Background

Many people in the developing world live in places that are characterized by unhealthy living circumstances. This is the case in the downstream areas of many rivers in Southeast Asia, where waste from the factories and people of the upper and lower parts of the river is accumulating, causing water pollution and house flooding: e.g. the Mekong and Red River Deltas in Cambodia and Vietnam, Manila bay, and the Mae Klong river in Thailand [1–6]. The Ciliwung river in Jakarta on the island of Java in Indonesia is an example of such a situation. The river is the largest among 13 rivers flowing through Jakarta, at approximately 130 km in length, with a catchment area of 390 square km. The Ciliwung river is heavily polluted with heavy metal concentrations such as lead (Pb) and zinc (Zn) [6–8], nitrate (NO3), human enteric viruses, and *Escherichia coli* [9, 10]. Moreover, it is frequently flooded, with its yearly peak occurring in January and February. When the floods hit, higher contaminations of viruses and bacterial indicators are found in the floodwaters [11].

Notwithstanding these circumstances, at the time of this study, many people still lived next to the Ciliwung. Living in such a place with high health risks, inadequate infrastructure, unreliable water and electricity supplies, and regular floods, was often perceived by the inhabitants as an acceptably safe and normal part of everyday life [12, 13]. People used the river water for washing and defecating. The children played and swum with their playmates. The houses had bad sanitation and were overcrowded; cats and mice could be found frequently [14, 15]. Evidently, such living conditions were accompanied by increased risks of different diseases, such as fecal-oral contagion, infectious diseases, skin complaints, and diarrhea. Despite the conditions, the inhabitants were reluctant to move or to be relocated by the government to other parts of Jakarta where better living conditions were available. This apparent contradiction raises questions concerning their subjective health and quality of life, including life satisfaction and happiness.

As elsewhere, government plans have been implemented in Jakarta to improve the state of such rivers in order to prevent pollution and flooding. For the later evaluation of the impact of these plans upon the lives of the people involved, knowledge of their health status and quality of life is required. Hence the aims of the present investigation were: 1) to obtain data on the health status and quality of life of people living on the Ciliwung riverbank, and 2) to compare these features with those of: i) a matched control group consisting of people with similar demographic characteristics, ii) inhabitants of Jakarta in general, and iii) the norm scores for the general population of Indonesia. The comparison groups were chosen to identify: i) the potential contribution of the target group’s specific living circumstances to their health status and quality of life; ii) how the group’s results on these features compared to those of (a) the overall inhabitants of their metropolitan city Jakarta, and b) the Indonesian people in general.

## Methods

### Respondents

We conducted the survey in Bukit Duri, an administrative urban village of South Jakarta city directly adjacent to the Ciliwung river. The population of Bukit Duri in 2015 consisted of 9233 families encompassing 32,679 subjects [16]. Of these families, approximately 400 lived by the Ciliwung. The inclusion criteria for this group, which will be referred to as ‘Ciliwung’ in this manuscript, were the following: i) living by the Ciliwung river, ii) aged 17 years or more, iii) an adequate command of the Indonesian language Bahasa Indonesia. The interviewers were introduced by members of the non-profit organization ‘Ciliwung Merdeka’, which operates in the area. As no formal street plan existed, nor any detailed information about the number of inhabitants per house, respondents were invited after knocking on each door. Because of this sampling approach, it was difficult to count non-responders, as more than one person could have been living in a household. We were able to interview 204 respondents.

The data for the three comparison groups: the Indonesian general population (which will be referred to as ‘general population’), Jakarta sample (‘Jakarta’), and a comparable matched control group (‘matched control’) were selected from our larger study which focused upon the Indonesian general population, in which several questionnaires were tested in a face-to-face setting at the home/office of the interviewer or at the homes of the subjects [17]. This larger study implemented a multi-stage stratified quota sampling procedure to ensure the sample’s representativeness of the Indonesian general population, resulting in 1041 respondents being interviewed in the final analysis. The sample was similar to the Indonesian population with respect to: location (urban/rural), gender, age, level of education, religion, and ethnicity [17]. For Jakarta as a comparison group, all respondents from the larger study who lived in Jakarta were included (*n* = 305). For the control group, we matched every respondent from the Ciliwung group with a respondent from the general population group with respect to their gender, age group, level of education, and monthly income. When there was more than one match for a respondent from of the Ciliwung population, a subject was randomly chosen from the possible matches.

### Procedure

The study was approved by the Health Research Ethics Committee, YARSI University, Jakarta. We hired four final year bachelors’ degree students at the YARSI University Faculty of Psychology as interviewers. All interviewers were trained by two of the authors at a half-day workshop concerned with the research project itself, the questionnaires, and the interview technique. The interviews were held at the homes of the respondents. Before they participated in the study, interviewers asked the respondents to read and sign informed consent forms. Respondents were encouraged to read the questionnaire by themselves, but if they had difficulty in reading: i.e. if they were illiterate, had low education levels, or eyesight problems, the interviewers would help them by reading aloud an item and asking them to indicate the answer in the questionnaire. Each respondent received a mug specifically designed for the study as a token of appreciation.

### Measures

Background and demographic characteristics of each respondent were obtained utilizing a questionnaire including questions about the respondent’s gender, age, ethnicity, education, religion, income, and marital status.

The health status of the respondents was measured by the official EQ-5D-5L Bahasa Indonesia version provided by the EuroQol Group. This translation of EQ-5D-5L was produced using a standardized translation protocol [18] and has proven to be valid and reliable in many countries [19–22] including in Indonesian population samples [23, 24]. The EQ-5D-5L is a generic HRQOL instrument which consists of two parts: i) five dimensions (mobility, self-care, usual activities, pain/discomfort, anxiety/depression), each of which can take one of five responses (no problems, slight problems, moderate problems, severe problems, and unable/extreme problems), and ii) the EQ Visual Analogue Scale (EQ-VAS), which records the respondent’s self-rated health on a 20 cm vertical visual analogue scale with endpoints labelled “the best health you can imagine” and “the worst health you can imagine” [25].

Quality of life was measured by the Indonesian version of WHOQOL-BREF, which is an abbreviated 26-item version of WHOQOL-100 that assesses four major domains: physical, psychological, social relationships, and environment. Each item is rated using a 5-point Likert scale with varied wording on each scale depending on the item (for example 1 = very dissatisfied to 5 = very satisfied). The scores are then transformed into a linear scale between 0 and 100, with 0 being the least favorable quality of life and 100 being the most favorable [26, 27]. The WHOQOL-BREF has been proved valid in a variety of contexts, and across many health conditions in many countries [28–32], including in Indonesia [33]. In line with the manual of the English version of WHOQOL-BREF [27] we chose to apply a time-frame of 4weeks, and our version was acknowledged by the WHO as the revised official Bahasa Indonesia version. We used the self-administered paper-based WHOQOL-BREF for this study. The Indonesian version of WHOQOL-BREF is available and has been proven as a valid and reliable questionnaire to be used in Indonesia [33] .

In addition, we measured the respondents’ personal happiness and life satisfaction. Personal happiness was assessed with the Happiness Thermometer, an 11-point scale for the assessment of happiness: during today, over the past month, and for life as a whole. The scale was graphically represented by 11 smileys presented horizontally, ranging from 0, represented by a ‘sad smiley’, to 5, represented by a neutral smiley, to 10, represented by a happy smiley. A similar measure showed good test-retest reliability, significant convergent validity coefficients, and the ability to distinguish small differences in happiness [34–36]. For this study’s sample, the internal consistency of the Happiness Thermometer scale was 0.78.

Life satisfaction was assessed with Cantril’s Self-Anchoring Striving Scale [37]. Participants were presented an 11-step vertical ladder, where the bottom step was marked with 0, the worst life possible, and the last step with 10, the best possible life. Participants were asked to assess satisfaction with their life at three time-points: now, 5 years ago, and 5 years from now. This measure is frequently used in surveys such as the Gallup World Poll [38]. The internal consistency of the Cantril’s Self-Anchoring Striving Scale in the present sample was 0.74.

Finally, we were interested in how the people of Ciliwung, who lived in a poor area of Jakarta, perceived their family’s financial situation given their relatively low incomes. We asked the following question: “We would like to know how you perceive your family’s financial situation. On the scale below, which number is the best reflection of your family’s financial situation now?” Then a 10-point horizontal VAS scale ranging from 0 (‘the poorest you can imagine’) to 10 (‘the richest you can imagine’) was presented for the respondents to choose.

The cultural adaptation of the questionnaires was conducted following guidelines from Guillemin [39] which consist of: forward translation, backward translation, committee review, and pre-testing. The EQ-5D-5L and WHOQOL-BREF were available in Bahasa Indonesia versions, provided by the EuroQol Group and World Health Organization, respectively. The Happiness Thermometer and Cantril’s Self-Anchoring Striving Scale were translated into Bahasa Indonesia by two native Indonesian speakers, backward translated into English by a native English speaker, and the study team held a meeting to check on the equivalence of the two translations. A pilot study of 46 inhabitants of Ciliwung was conducted to test the feasibility of the questionnaires and revision was subsequently undertaken based on the respondents’ input. The inclusion of the family’s financial situation scale was based on this pilot study.

### Analysis

The demographic characteristics were described as percentages within the subgroups in each sample: i.e. gender, age group, education level, ethnicity, religion, monthly income and marital status. For the self-reported health profile obtained from EQ-5D-5L, we calculated the percentages of respondents for each level of each dimension. We then combined level 2 (slight problems) through to level 5 (unable/extreme problems) into ‘any problems’ and presented this along with level 1 (no problems). The proportions of the Ciliwung and the three comparison groups’ respondents who reported any problems were compared using the Chi-square test. The EQ-5D-5L health states were converted into a single index score using the Indonesian value set [17] and EQ-VAS was scored by transforming the 20 cm VAS into a 0–100 scale [40]. Mean and standard deviation were calculated for each different domain of WHOQOL-BREF, and for visual analogue scales of perceived happiness, life satisfaction, and financial situation.

For the comparison between the Ciliwung sample and the three other groups of the domains of each variable: health status (EQ-VAS and index score), quality of life (physical, psychological, social relationships and environment domains from WHOQOL-BREF), personal happiness (today, over the past month, and whole life), life satisfaction (now, 5 years ago, and 5 years from now), and financial situation, we applied t-tests if the data was normally distributed or the Wilcoxon rank-sum test if not normally distributed. Normality was tested using the Shapiro-Wilk test. We also applied one-way MANOVA to test the difference between groups across each outcome variable’s domains simultaneously: health status, quality of life, happiness, and life satisfaction. The groups - Ciliwung, matched control, Jakarta, general population - served as the predictors. Further multiple linear regression analysis was carried out to evaluate the group differences when controlling for socio-demographic variables: gender, age, education, monthly income, ethnicity, religion, and marital status. Additional multiple linear regression analyses were conducted to evaluate the group differences in the average scores of the three time-points on the Happiness Thermometer and on Cantril’s Self-Anchoring Striving Scale when controlling for socio-demographic variables. *P* < 0.05 was considered significant. To determine the magnitude of the differences we calculated the effect size using Cohen’s d and applied the criteria from Cohen for the interpretation: 0.2–0.5 = small, 0.5–0.8 = medium, > 0.8 = large difference [41].

## Results

### Demographic characteristics of respondents

As could be expected, the Ciliwung group did not differ from the matched controls in each of the demographic characteristics (see Table 1). Compared to the general population and Jakarta samples, the Ciliwung group did not differ in age and gender. On the other hand, the group had on average a lower education, monthly income, and percentage of single/divorced persons compared to the general population and Jakarta samples. The majority of the Ciliwung group had a Batavian ethnic and Islam background, with similar percentages to the Jakarta group.

**Table 1:** Comparison of Demographic Characteristics of Ciliwung sample with matched control group, Jakarta group, Indonesian general population group

Characteristic	Level	Ciliwung *N* = 204		Matched control *N* = 204		Jakarta *N* = 305		General population *N* = 1041	
		n	%	n	%	n	%	n	%
Age	17–30 years	80	39.2	80	39.2	105	34.4	412	39.6
	31–50 years	88	43.1	88	43.1	151	49.5	434	41.7
	> 50 years	36	17.7	36	17.7	49	16.1	195	18.7
Gender	Male	102	50.0	102	50.0	137	44.9	521	50.1
	Female	102	50.0	102	50.0	168	55.1	520	49.9
Level of Education (highest)	Primary school or lower	71	34.8	68	33.3	74	24.3^*^	334	32.1
	High school	129	63.2	132	64.7	190	62.3	546	52.4^*^
	College/University	4	2.0	4	2.0	41	13.4^*^	161	15.5^*^
Income/month (Euro)	< 500 K IDR (< 30)	108	52.9	108	52.9	94	30.8^*^	507	48.7
	500-2500 K IDR (30–150)	68	33.3	68	33.3	109	35.8	354	34.0
	2500-5000 K IDR (150–300)	26	12.8	26	12.8	79	25.9^*^	130	12.5
	5000-10,000 K IDR (300–600)	2	1.0	2	1.0	19	6.2^*^	40	3.8^*^
	> 10,000 K IDR (> 600)	0	0.0	0	0.0	4	1.3	10	1.0
Ethnicity	Batavian	99	48.5	34	16.6^*^	109	35.7^*^	110	10.6^*^
	Javanese	55	27.0	81	39.7^*^	82	26.9	433	41.6^*^
	Sundanese	39	19.1	34	16.7	8	2.6^*^	198	19.0
	Sumatran	6	2.9	25	12.3^*^	64	21.0^*^	129	12.48
	Other	5	2.5	30	14.7^*^	42	13.8^*^	171	16.4^*^
Religion	Islam	203	99.5	203	99.5^*^	292	95.7^*^	911	87.5^*^
	Christian	1	0.5	1	0.5^*^	7	2.3	99	9.5^*^
	Others	0	0.0	0	0.0	6	2.0^*^	31	3.0^*^
Marital Status	Married	154	75.5	128	62.7^*^	196	64.3^*^	619	59.5^*^
	Divorced/Single	50	24.5	76	37.3^*^	109	35.7^*^	422	40.5^*^

^*^Difference of proportion between Ciliwung and corresponding groups: control, Jakarta, general population, statistically significant (*p*-value< 0.05)

### Comparison between groups

Table 2 shows that by comparison with the matched control group, the Ciliwung group had significantly lower scores for the physical domain of quality of life (WHOQOL-BREF) and ‘feeling happy today’. However, the group scored significantly higher on life satisfaction for all three time points and perceived financial situation. Self-perceived health measured with EQ-5D-5L (EQ-VAS) showed the opposite direction to that measured by WHOQOL-BREF: Ciliwung respondents reported significantly higher (more favorable) scores than the matched control group. Note that most effect sizes were small, except that for the physical domain of WHQOL-BREF, which was moderate.

**Table 2:** Health status and quality of life of Ciliwung sample in comparison with groups: matched control, Jakarta, general population

Aspect	Dimension	Ciliwung		Matched controls			Jakarta			General population		
		Mean	SD	Mean	SD	ES^a^	Mean	SD	ES	Mean	SD	ES
Health status	EQ-VAS	81.74	15.39	78.85^*^	13.24	0.20	77.50^*^	13.15	0.3	79.41^*^	14.03	0.16
	Index score	0.91	0.15	0.91	0.11	0.00	0.90	0.12	0.09	0.91	0.11	0.01
Quality of life	Physical	63.31	11.56	69.66^*^	10.60	0.57	68.77^*^	11.23	0.48	69.23^*^	11.50	0.52
	Psychological	64.24	14.86	66.14	13.69	0.13	65.77	12.77	0.11	66.74^*^	12.89	0.19
	Social	59.48	14.78	62.25	14.9	0.19	63.33^*^	14.28	0.27	63.13^*^	14.41	0.25
	Environment	53.62	14.21	55.94	13.88	0.17	58.02^*^	12.50	0.33	58.49^*^	13.41	0.36
Happiness	Today	6.75	2.28	7.26^*^	1.79	0.25	7.31^*^	2.05	0.26	7.35^*^	1.84	0.31
	Last month	6.48	2.26	6.90^*^	1.98	0.20	7.09^*^	2.14	0.28	7.05^*^	1.94	0.28
	Whole life	6.94	2.11	7.28	1.73	0.18	7.56^*^	1.86	0.32	7.37^*^	1.78	0.23
Life satisfaction	Now	7.01	2.11	6.34^*^	1.84	0.34	6.51^*^	1.87	0.26	6.47^*^	1.89	0.28
	5 years ago	6.20	2.36	5.69^*^	2.03	0.23	5.88	2.18	0.14	5.79^*^	2.06	0.19
	5 years later	8.78	1.80	8.24^*^	1.76	0.31	8.50^*^	1.58	0.17	8.29^*^	1.71	0.29
Financial condition	Now	5.70	1.91	4.99^*^	1.73	0.39	5.45	1.53	0.15	5.23^*^	1.83	0.25

^*^Differences between Ciliwung mean and means of corresponding groups: matched control, Jakarta, general population, statistically significant (*p*-value< 0.05)

^a^Effect size based on Cohen’s d

Compared to the Jakarta respondents, the Ciliwung group reported significantly lower scores on three quality of life domains (physical, social, and environmental**),** and on personal happiness for all time points. However, the group’s scores on their perceived health status (EQ-VAS) and on their current and future life satisfaction, were significantly higher than the Jakarta group. The effect sizes were small in all comparisons.

A similar picture was shown when comparing the Ciliwung group and the general population: Ciliwung respondents scored lower on quality of life and happiness, but higher on health status (VAS), life satisfaction, and perceived financial situation. Most effect sizes were small, with the exception of that for the physical domain of WHQOL-BREF, which was moderate.

Exploring health status in more detail, the percentage of Ciliwung respondents who reported ‘no problem’ on all dimensions of EQ-5D-5L (‘11111’) was significantly higher than that of the comparison groups, as can be seen in Table 3. When we looked at the proportions of ‘any problems’ (levels 2–5) reported per dimension, the Ciliwung group had significantly less anxiety/depression than each of the comparison groups. For the other four dimensions, the proportions of ‘any problems’ were similar.

**Table 3:** EQ-5D-5L Self-reported health profiles: four group samples (%)

Sample		Mobility		Self-Care		Usual Activity		Pain/Discomfort		Anxiety/Depression		Reported ‘11111’^a^
	N	No	Any	No	Any	No	Any	No	Any	No	Any	
Ciliwung	204	90.20	9.80	97.06	2.94	90.20	9.80	64.22	35.78	84.31	15.69	55.39
Controls	204	91.67	8.33	98.53	1.47	87.75	12.25	60.78	39.22	68.63	31.37^*^	44.12^*^
Jakarta	305	88.52	11.47	98.36	1.64	84.92	15.08	59.67	40.32	63.28	36.72^*^	37.70^*^
General	1041	92.03	7.97	98.08	1.92	89.15	10.86	60.61	39.39	66.09	33.91^*^	43.70^*^

^*^Difference between proportions of respondents in the specific dimensions between Ciliwung and corresponding group statistically significant (*p*-value< 0.05)

^a^Percentage of respondents who reported no problems (level 1) on all five dimensions of EQ-5D-5L

The MANOVA analysis demonstrated statistically significant differences between the Ciliwung group and the matched control group in quality of life and life satisfaction (Wilks lambda 0.915 and 0.965, respectively), but not in health status and happiness. Further, the Ciliwung group was significantly different from the other groups in each of the outcome variables (Wilks lambda between 0.936 and 0.986), with the exception of health status (where there was no significant difference with the general population).

When controlling for socio-demographic factors: i.e. gender, age, education, monthly income, ethnicity, religion, and marital status (see Table 4), the outcomes were similar overall to those which were uncontrolled (see Table 2). When we averaged the respondents’ responses at the three different time points on the happiness and life satisfaction scales, the results were similar to those shown in Table 4: the Ciliwung group was significantly different from the other groups in happiness and life satisfaction scores.

**Table 4:** Linear multiple regression coefficients for quality of life, health status, happiness, life satisfaction, and financial condition with groups and demographics as independent variables

		Location	Gender^a^	Age	Education^b^		Income^c^		Ethnicity^d^				Religion^e^		Marital status^f^	Constant
		Ciliwung	Male		Middle	High	500-2500 K	> 2500 K	Sundanese	Bataknese	Batavia	Others	Christian	Others	Married	
Health Status	EQ-VAS															
	vs Control^g^	2.88^*^	–	–	–	–	–	–	–	–	–	–	–	–	–	78.85^*^
	vs Jakarta	5.81^*^	2.94^*^	−0.11^*^	−1.48	1.62	− 0.64	2.91	−6.31^*^	−4.84^*^	−7.22^*^	−4.23	9.27	−5.48	−1.69	85.45^*^
	vs General	4.47^*^	2.26^*^	−0.10^*^	2.13^*^	5.39^*^	−1.74	1.56	2.18	−3.38^*^	−5.27^*^	0.66	−0.12	−6.09^*^	0.83	80.53^*^
	Index score															
	vs Control	0.001	–	–	–	–	–	–	–	–	–	–	–	–	–	0.912^*^
	vs Jakarta	0.025^*^	0.026^*^	−0.003^*^	−0.007	− 0.004	0.008	0.032	−0.044^*^	− 0.012	− 0.029^*^	− 0.016	0.082	0.014	0.029^*^	0.985^*^
	vs General	0.010	0.024^*^	−0.002^*^	0.001	0.009	−0.001	0.021^*^	−0.022^*^	− 0.035^*^	− 0.037^*^	− 0.017	0.016	−0.034	0.024^*^	0.953^*^
Quality of life	Physical															
	vs Control	−6.36^*^	–	–	–	–	–	–	–	–	–	–	–	–	–	69.66^*^
	vs Jakarta	−3.84^*^	2.32^*^	−0.10^*^	1.42	0.32	−0.13	2.27	−3.16	3.06	−0.26	0.04	5.12	−3.03	−0.29	69.34^*^
	vs General	−4.87	1.95	−0.14	0.15	−0.30	0.29	2.46	−3.15^*^	0.48	−1.60	0.85	1.27	−1.54	0.21	73.18^*^
	Psychological															
	vs Control	−1.91	–	–	–	–	–	–	–	–	–	–	–	–	–	66.14^*^
	vs Jakarta	0.24	1.18	−0.14^*^	− 0.08	−3.64	2.75	6.74^*^	−1.01	2.43	−1.66	0.57	1.96	−6.50	0.71	67.39^*^
	vs General	−0.85	1.81	−0.11	1.01	0.85	0.40	4.19	−2.70^*^	0.11	−3.41^*^	1.20	1.89	−4.25	1.13	68.13^*^
	Social															
	vs Control	−2.78	–	–	–	–	–	–	–	–	–	–	–	–	–	62.26^*^
	vs Jakarta	− 2.76	1.59	−0.08	1.14	2.23	−0.15	4.28^*^	−0.42	0.96	0.83	0.30	0.89	1.79	1.83	61.36^*^
	vs General	−3.07	1.91	−0.11	2.63	3.93	− 0.10	2.83	−0.85	1.12	0.40	4.87^*^	1.08	−5.51^*^	3.08^*^	61.23^*^
	Environmental															
	vs Control	−2.33	–	–	–	–	–	–	–	–	–	–	–	–	–	55.94^*^
	vs Jakarta	−2.24	−0.02	−0.08	1.38	1.55	−0.13	5.54^*^	−1.47	4.17^*^	1.37	−1.31	0.10	10.26	0.36	56.64^*^
	vs General	−3.27	−0.42	− 0.08	1.29	3.32	−0.51	4.52	−1.98	0.89	0.01	4.35^*^	1.52	2.73	−0.71	59.60^*^
Happiness	Today															
	vs Control	−0.51^*^	–	–	–	–	–	–	–	–	–	–	–	–	–	7.26^*^
	vs Jakarta	−0.30	−0.19	0.00	0.34	−0.03	0.37	0.99^*^	0.25	0.32	0.01	−0.10	−0.60	0.90	−0.28	6.98^*^
	vs General	−0.48^*^	− 0.21	− 0.01	0.42^*^	0.52^*^	− 0.24	0.29	0.13	0.22	−0.12	0.26	0.19	−0.53	0.16	7.25^*^
	Last month															
	vs Control	−0.42^*^	–	–	–	–	–	–	–	–	–	–	–	–	–	6.90^*^
	vs Jakarta	−0.53^*^	−0.14	− 0.01	0.08	− 0.09	0.28	0.45	0.62	0.58	0.17	0.15	−0.23	0.63	0.29	6.70^*^
	vs General	−0.50^*^	−0.10	0.00	0.20	0.49^*^	−0.26	0.04	0.24	0.35	0.09	0.29	0.52^*^	−0.25	0.34^*^	6.69^*^
	Whole life															
	vs Control	−0.34	–	–	–	–	–	–	–	–	–	–	–	–	–	7.28^*^
	vs Jakarta	−0.47^*^	0.05	−0.01	0.34	−0.22	0.08	0.42	0.31	0.65^*^	0.03	−0.02	0.18	0.77	−0.11	7.27^*^
	vs General	−0.38^*^	−0.13	− 0.01	0.45^*^	0.61^*^	−0.21	0.18	0.07	0.39^*^	0.05	0.22	0.06	−0.68	0.19	7.11^*^
Life Satisfaction	Today															
	vs Control	0.68^*^	–	–	–	–	–	–	–	–	–	–	–	–	–	6.34^*^
	vs Jakarta	0.64^*^	0.11	−0.01	−0.50^*^	−0.17	0.11	0.47	−0.06	0.44	−0.11	−0.27	− 0.63	0.20	0.10	6.76^*^
	vs General	0.62^*^	−0.14	0.00	0.34^*^	0.44^*^	−0.35^*^	0.09	0.51^*^	0.45^*^	0.09	0.68^*^	0.00	−0.25	0.30^*^	6.02^*^
	5 years ago															
	vs Control	0.52^*^	–	–	–	–	–	–	–	–	–	–	–	–	–	5.69^*^
	vs Jakarta	0.52^*^	0.17	−0.02^*^	−0.54^*^	−0.25	− 0.08	0.00	−1.00^*^	0.52	0.15	−0.18	−1.09	0.32	−0.06	6.81^*^
	vs General	0.34	−0.06	0.00	0.35^*^	0.53^*^	−0.50^*^	−0.33	0.10	0.39	0.38^*^	0.29	−0.04	0.09	0.09	5.54^*^
	5 years later															
	vs Control	0.54^*^	–	–	–	–	–	–	–	–	–	–	–	–	–	8.24^*^
	vs Jakarta	0.43^*^	−0.10	−0.01	−0.07	0.32	0.35^*^	0.23	0.21	0.15	0.18	0.54	0.25	−0.80	−0.19	8.54^*^
	vs General	0.51^*^	−0.10	−0.01	0.55^*^	0.63^*^	−0.23^*^	−0.07	0.50^*^	0.45^*^	0.37^*^	0.70^*^	0.09	−0.53	0.07	7.98^*^
Financial	Financial situation															
	vs Control	0.71^*^	–	–	–	–	–	–	–	–	–	–	–	–	–	4.99^*^
	vs Jakarta	0.48^*^	0.17	0.00	−0.04	0.63	−0.12	0.41	−0.04	0.30	−0.21	−0.36	− 0.38	1.19	− 0.21	5.48^*^
	vs General	0.63^*^	−0.24^*^	0.00	0.72^*^	1.16^*^	0.00	0.57^*^	0.75^*^	0.63^*^	0.21	0.49^*^	0.00	0.58	0.04	4.46^*^

^*^p-value < 0.05

^a^Female is the reference group

^b^Basic education level: primary school and below is the reference group

^c^Monthly income less than 500 K IDR is the reference group

^d^Javanese is the reference group

^e^Islam is the reference group

^f^Single/divorced is the reference group

^g^For comparison between Ciliwung and reference group, univariate linear regression was used

## Discussion

Our findings are the first with respect to the quality of life and health status of people living in uncertainty due to floods, pollution, and possible relocation. These people lived on the banks of the Ciliwung river in Jakarta, Indonesia. A demographically-matched control group was utilized in the study. We found that the Ciliwung respondents reported lower quality of life on the physical domain but experienced higher health status (EQ-VAS) than the matched controls. Further, Ciliwung respondents perceived themselves as less happy but more satisfied with their lives than the controls. Their differences with the Jakarta and general population samples were comparable. In addition, they perceived themselves as richer than people living in Jakarta and the general population, although their actual incomes were lower.

The lower level of physical health in the Ciliwung group was understandable given the unhealthy environment. However, the better health status and life satisfaction compared to the other three groups, illustrated by a higher EQ-VAS score, fewer anxiety/depression problems and higher life satisfaction scores, was surprising considering the living environment, which was highly polluted and often flooded, the lower income, and the smaller houses. This finding also appears contradictory to a number of investigations of health status in general populations, e.g. in Indonesia [42], Singapore [43], Sri Lanka [44], and South Australia [45], where groups with lower education levels and incomes usually reported lower health status. It should be noted that there is no information from these studies on whether or not their general population respondents were living in polluted river areas. Moreover, the Ciliwung group life satisfaction score was higher than the average Indonesian score in the World Happiness Report 2017 published by the United Nations [46]. Notwithstanding this, the people of the Ciliwung group reported themselves as being less happy compared to the three comparison groups, which was more in line with what we expected.

Several investigations reported that people living in poor and regularly flooded areas of Jakarta acknowledged that they faced many problems: e.g. poverty, lack of facilities, space limitations, and regular floods. All these problems put a severe burden on the inhabitants’ health, emotional, security, and economic circumstances [13, 47, 48]. However, the present study found positive outcomes in terms of better self-reported health status and life satisfaction regardless of their poor living conditions. Several possible explanations can be identified and are also mentioned in the literature, often based on qualitative research: adaptation, relative comparison**s**, and social capital. First, the people living on the banks of the Ciliwung river had learned to cope with certain life conditions; they considered the yearly floods as a normal part of everyday life to which they had become accustomed. These people knew what to do during floods, how to protect their belongings, and how to recover after a flood. As a close community, they developed physical (e.g. raising house levels) and non-physical (a communal work system to minimize the effect of a flood, the re-use of surviving material after a flood) responses to floods, in other words, they became resilient [12, 47–49]. Second, the Ciliwung respondents might have been comparing their life situations with those of their nearest neighbors, with similar low levels of income and life conditions, which might have prevented them from becoming envious, whilst the comparison group respondents might have had a broader range of incomes in their neighborhoods. Third, these people had lived there for generations amongst those they had known for life, often with similar ethnicity and religion. They knew their neighbors, which meant: they could depend upon them in times of distress, they had quick access to formal and informal job opportunities, and support in times of lifecycle events such as marriage, sickness, and death [12, 49]. Moreover, they developed community-based organizations that helped them to organize both formal and informal strategies to cope with the uncertainty of policies concerning eviction and yearly floods [50]. This ‘social capital’ might have raised their levels of life satisfaction. Some members of the community who succeeded in improving their economic situation and relocated to a middle-class neighborhood returned after a short time because they: (i) missed the strong social cohesion amongst their former neighbors, (ii) realized that the cost of living in their poor former community was cheaper than in their new neighborhood, and (iii) acknowledged the advantage of the strategic location of their previous neighborhood [49].

Several limitations of this study should be considered. First, the data was collected at a time of escalation of tension between the people of Kampung Pulo and the government of Jakarta, i.e. in the area across the river from Bukit Duri, concerning the possibility of relocation to some large blocks of flats provided by the Jakarta government. The plan was to relocate people from Bukit Duri who lived on the riverbank after the relocation of Kampung Pulo was finished. Remarkably, this did not lead to an increased prevalence of reported anxiety or depression compared with the other groups. Indeed, it is also difficult to judge if and how the possibility of relocation in the near future may have had an impact on the respondents’ subjective well-being. In the event, a month after completion of the data collection, the inhabitants of Bukit Duri received a final letter from the government announcing the exact date of their relocation, which was realized several months later. Their former homes were demolished in order to improve the river’s condition.

Second, respondent recruitment might raise questions about the objectivity/representativeness of the study sample since we asked non-governmental organization officers to introduce us to the community. This might have entailed some bias in terms of interdependent data collection. However, we matched the proportions of the Bukit Duri population with respect to gender, age, and level of education with a control group. As can be seen in Table 1, we succeeded in constructing a representative sample.

## Implications

Our results have some implications for future studies. During the writing of this manuscript, the relocation of the respondents living on the banks of the Ciliwung river in Bukit Duri to large blocks of flats was accomplished by the government of Jakarta. Considering the findings of lower levels of physical health and happiness of the Ciliwung respondents, relocation to a better living environment might be expected to have improved these aspects of their life. However, it would be interesting to follow up whether living in large blocks of flats, which from a distance might be considered as providing better living conditions, would indeed affect health status and life satisfaction in a positive way. Furthermore, it would be interesting to find out if and how these changes: geographic location, living conditions, and dwelling in flats instead of houses, would impact upon the dynamic inter-relationships within the community, their social capital, and community resilience. Future studies combining quantitative and qualitative methods could obtain a comprehensive picture of the effects of relocation on the people involved. A quantitative study could be undertaken by repeating the measurement of HRQOL in the current research population with respect to happiness, life satisfaction, and perceived economic circumstances in their new living environment and to compare these data with the previous data before their relocation. A qualitative study could be accomplished by utilizing in-depth interviews and observations of the respondents, focusing on their experiences of being relocated. Results from the present and future studies could be used by government, local and national, when developing policies related to people living in unhealthy areas, such as on the riverbank of a polluted river.

## Conclusion

People living on a polluted and flooding riverbank in a large city showed a lower quality of life, particularly physical, and fewer feelings of happiness, than a comparable group that did not live there. The differences were small overall. Moreover, the people living on the riverbank perceived themselves to be better in terms of health status in general, life satisfaction, and financial situation. Hence the relocation to better housing and an improved environment might be expected to improve their physical health and quality of life, but not necessarily their satisfaction with life and the perception of their financial circumstances.

## Abbreviations

EQ-5D-5L: Five-level EuroQol five-dimensional questionnaire
QoL: Quality of life
WHOQOL-BREF: World Health Organization Quality of life BREF
